# Differences in the Faecal Microbiome in *Schistosoma haematobium* Infected Children vs. Uninfected Children

**DOI:** 10.1371/journal.pntd.0003861

**Published:** 2015-06-26

**Authors:** Gemma Louise Kay, Andrew Millard, Martin J. Sergeant, Nicholas Midzi, Reggis Gwisai, Takafira Mduluza, Alasdair Ivens, Norman Nausch, Francisca Mutapi, Mark Pallen

**Affiliations:** 1 Microbiology and Infection Unit, Division of Translational and Systems Medicine, Warwick Medical School, University of Warwick, Gibbet Hill Campus, Coventry, West Midlands, United Kingdom; 2 National Institute of Health Research, Causeway, Harare, Zimbabwe; 3 Murewa District Hospital, Murewa, Zimbabwe; 4 Department of Biochemistry, University of Zimbabwe, Mount Pleasant, Harare, Zimbabwe; 5 Insitute of Immunology and Infection Research, Centre for Immunology, Infection and Evolution, School of Biological Sciences, University of Edinburgh, Ashworth Laboratories, King’s Buildings, Edinburgh, United Kingdom; University of Melbourne, AUSTRALIA

## Abstract

**Background:**

Several infectious diseases and therapeutic interventions cause gut microbe dysbiosis and associated pathology. We characterised the gut microbiome of children exposed to the helminth *Schistosoma haematobium* pre- and post-treatment with the drug praziquantel (PZQ), with the aim to compare the gut microbiome structure (abundance and diversity) in schistosome infected vs. uninfected children.

**Methods:**

Stool DNA from 139 children aged six months to 13 years old; with *S*. *haematobium* infection prevalence of 27.34% was extracted at baseline. 12 weeks following antihelminthic treatment with praziqunatel, stool DNA was collected from 62 of the 139 children. The 16S rRNA genes were sequenced from the baseline and post-treatment samples and the sequence data, clustered into operational taxonomic units (OTUs). The OTU data were analysed using multivariate analyses and paired T- test.

**Results:**

Pre-treatment, the most abundant phyla were Bacteroidetes, followed by Firmicutes and Proteobacteria respectively. The relative abundance of taxa among bacterial classes showed limited variation by age group or sex and the bacterial communities had similar overall compositions. Although there were no overall differences in the microbiome structure across the whole age range, the abundance of 21 OTUs varied significantly with age (FDR<0.05). Some OTUs including *Veillonella*, *Streptococcus*, *Bacteroides* and *Helicobacter* were more abundant in children ≤ 1 year old compared to older children. Furthermore, the gut microbiome differed in schistosome infected vs. uninfected children with 27 OTU occurring in infected but not uninfected children, for 5 of these all Prevotella, the difference was statistically significant (p <0.05) with FDR <0.05. PZQ treatment did not alter the microbiome structure in infected or uninfected children from that observed at baseline.

**Conclusions:**

There are significant differences in the gut microbiome structure of infected vs. uninfected children and the differences were refractory to PZQ treatment.

## Author Summary

The role of the gut microbiome in host health is becoming clearer with better characterisation of the nutritional, biochemical and immunological function of the microbes. However, to date, there is a paucity of studies describing/ investigating the role of the gut microbiome in infection, pathology and acquired immunity in children exposed to helminths, in sub-Saharan Africa. We determined how the diversity and abundance of different gut bacteria taxonomic groups differ with age, sex and infection with the helminths parasites causing bilharzia/schistosomiasis in children aged 6 months -13 years. The parasites are controlled by treatment with the antihelminthic praziquantel (PZQ), which is associated with diarrhoea within the first 24 hours of treatment in some children. We therefore also investigated the effects of PZQ treatment on the gut microbiome of the children 12 weeks post-PZQ treatment. The composition of the gut microbiome changed with age within the 0–3 year age range and also differed significantly in abundance of the different taxonomic groups between infected and uninfected children prior to treatment with PZQ. However, the pre-treatment microbiome structure was not altered by antihelminthic treatment of infected or uninfected children. Further studies are required to investigate causality and mechanisms of interaction.

## Introduction

The importance of the gut microbiome in host health is becoming increasingly clear as studies characterise the nutritional, biochemical and immunological function of the microbes. Several studies have highlighted that significant alteration of the gut microbiome (dysbiosis) can influence susceptibility to non-infectious diseases [1], while experimental studies have described both beneficial and detrimental effects of gut microbes on host health and response to pathogens and therapeutics [2,3]. Recently we reviewed the interactions and associations between helminth parasites and the gut microbiome in humans highlighting significant knowledge gaps [4]. In particular, there is a paucity of studies describing/investigating the role of the gut microbiome in infection, pathology and acquired immunity in children exposed to helminth infections. The most important helminth parasite in sub-Saharan Africa is *Schistosoma haematobium* which causes urogenital schistosomiasis, commonly known as bilharzia. Urogenital schistosomiasis significantly affects childhood health and development [5].

Helminth infections have been shown to affect non-parasite-specific host immune responses [6–9]. Our own studies have shown that both antibody [10] and cellular responses [11–13] are altered during *S*. *haematobium* infection, while our mechanistic studies have started to shown how schistosomes modulate the immune pathways (see for example [14,15]). Investigations of interaction between the gut microbiome (also known to play a central role in the development and homeostasis of the immune response) and helminth infection (susceptibility to infection and pathology) are still in their infancy. Work in the mouse model suggests that depletion of the gut bacteria results in a reduction in schistosome egg excretion, altered parasite specific immune responses and reduced inflammatory responses and gut pathology [16]. Such studies however, have yet to be conducted in the natural human schistosome host and in *S*. *haematobium*. Conversely, the effect helminths may have on the gut microbiome structure (and associated mechanisms) still need to be investigated in human populations, if we are to gain information to improve human health.

Currently potential mechanisms of how the gut microbiome can interact with schistosome infections are being investigated in experimental models of intestinal schistosomiasis, but there are no such studies published for *S*. *haematobium* and thus no indication of the potential mechanisms of interaction. The presence of *S*. *haematobium* eggs in the GIT [17] provides in theory, an opportunity for direct interaction between the parasites and the gut flora. Studies in Syrian hamsters [18], have shown shifts in the composition of the gut microbiota during helminth infection, while a recent study in humans reported that people infected with the intestinal helminths of the genus *Trichuris*, had greater gut bacteria species richness compared to uninfected people [19].

More likely, the interaction may occur at the systemic level. The gut microbiome has been linked to the systemic autoimmune disease rheumatoid arthritis [20], suggesting a systemic effect of gut bacteria on host health. Thus, gut bacteria may have a systemic effect on schistosome parasites residing in the bladder plexus, suggesting the possibility of interaction between schistosomes and the gut microbiome. Conversely, the already established helminth modulatory effects on the host immune system (for example [6–9]) may extend to influencing the structure of the gut microbiome.

In addition to changes to the gut microbiome associated directly with the parasites, the treatments used to control these infections may also have a significant impact on the gut bacteria abundance and diversity. Praziquantel (PZQ) is effective against all schistosome species affecting humans and is taken orally as a single dose, typically 40 mg/kg body weight. PZQ kills adult schistosome worms and reverses early schistosome-related pathology [17]. We and others have been investigating the short and long-term effects of PZQ treatment as part of studies on the safety and host health effects of PZQ treatment [21]. Diarrhoea is often reported within 24 hours of taking PZQ [22]. Diarrhoea is associated with gut microbiome dysbiosis but the effects of PZQ treatment on the gut microbiome have never been investigated [23]. Thus, the aims of this study were to determine if there were differences in the structure (diversity and abundance) of the faecal microbiome between children infected with *S*. *haematobium* and uninfected children and to investigate the effects of PZQ treatment on the structure of the gut microbiome.

## Materials and Methods

### Ethical statement

The study was conducted in the Murewa district in Zimbabwe (31°63’E; 17°52′S) in the Mashonaland East Province of Zimbabwe, where *S*. *haematobium* is endemic, as part of a larger study on paediatric schistosomiasis. The study area and populations are described in detail elsewhere [24]. Ethical approval was received from the Medical Research Council of Zimbabwe, and permission to conduct the study was obtained from the Provincial Medical Director. The study design, aims and procedures were explained in the local language, Shona, prior to enrolment. Children (six months-13 years old) from Chingwaru Primary school were enrolled into the project after written informed consent/assent was obtained from participants/guardians.

### Study area and population

The area is a high transmission area for *S*. *haematobium* according to the WHO classification [25] and was selected due to its low prevalence of *S*. *mansoni* and soil-transmitted helminths (STH) [26] to avoid confounding from the other helminth infections.

### Sample collection

Stool and urine specimens were collected in standard specimen collection bottles between 10 am and 12 noon, stored at ambient temperature and transported to the laboratory for processing. *S*. *haematobium* infection was diagnosed by microscopic examination of parasite eggs in urine processed by the standard urine filtration method [27] at baseline and at the 12 weeks post-treatment efficacy check survey. Stool samples were processed using the Kato-Katz method [28] and subsequently examined by microscopy for the diagnosis of *S*. *mansoni* and STHs, while a sample was also taken from the stool sample collected on the first sampling day and processed for DNA extraction. Children were classified as infected with *S*. *haematobium* parasites if at least one parasite egg was detected in any of the collected urine samples, and as infected with *S*. *mansoni* parasites if at least one parasite egg was detected in any of the collected stool samples. After sample collection, all compliant participants were treated by the attending physician with the standard dose of 40 mg/kg body weight PZQ.

### Inclusion criteria

To be included in the cross-sectional study, participants had to meet the following criteria: (1) be lifelong residents of the study area, (2) have no prior history of antihelminthic or antibiotic treatment (assessed by questionnaire administered to participants/guardians), (3) had provided at least two urine and at least two stool samples (over three consecutive days) for parasitological examination and stool DNA, (4) be negative for *S*. *mansoni* and any STH (no children were excluded on this criteria as all were negative for these infections). 139 children met these criteria and were included in the cross-sectional study. To be included in the longitudinal aspect of the study, children had to meet the following additional criteria: (1) have been offered treated with PZQ, (2) had provided at least two urine and two stool samples for parasitological examination and stool DNA 12 weeks after PZQ treatment and (3) have to have cleared infection 12 weeks after PZQ treatment if treated. 62children met these additional criteria and were eligible for inclusion in this aspect of the study.

### DNA extraction

Stool DNA was extracted from 200 mg of stool using the QIAamp DNA stool extraction kit (Qiagen, UK) following the manufacturer’s protocol for pathogen detection. Extracted DNA was frozen and shipped to University of Edinburgh where samples were defrosted, quantified using Qubit 2 (Invitrogen), diluted and aliquoted. Aliquoted samples were shipped to the University of Warwick for high-throughput sequencing.

### High-throughput sequencing of V3-V4 16S rRNA gene fragments

Samples were diluted to 2 ng/μl and the V3-V4 region of the 16S rRNA gene was amplified including sequencing barcodes (primers detailed in S1 Table) with extensor master mix (Fisher Scientific) using the following cycle conditions: 94°C for 3 min; 25 cycles of 94°C for 30 s, 55°C for 30 s, 68°C for 1 min; 68°C 5 min. Amplicon libraries were purified using Ampure XP magnetic beads (Beckman Coulter) and quantified using the broad range dsDNA Qubit assay (Fisher Scientific). Each amplicon library was diluted to 6 nM and 10 μl of each dilution pooled to generate two sets of 95 samples and one set of 44 (including 9 repeats from sets 1 and 2 which generated <30,000 reads). Sequencing was performed on the Illumina MiSeq platform using paired-end 2 x 250 bp protocol with V2 reagents.

### Data analysis

Reads were demultiplexed according to the truseq barcode using a custom JAVA script, which used the barcode in read 1 (100% match), prior to removal of the barcode from read 2. Reads were then joined with USEARCH, allowing for a maximum of 3 mismatches, the resulting sequences were de-replicated and clustered following the USEARCH framework. Clusters containing less than four sequences were removed.

This resulted in a total data set of approximately 19 million reads. The data was processed using the software package Quantitative Insights Into Microbial Ecology (QIIME) [29]. Taxonomy was assigned to 16S rRNA gene sequences using the default algorithm in QIIME (RDP classifer). Prior to further analyses, samples were rarefied to account for variation in the number of sequences per sample. Alpha diversity indexes were calculated in QIIME from rarefied samples using the Shannon index [29]. Beta diversity was calculated using weighted and unweighted UniFrac. Principal coordinates analysis was then performed on all rarefied distance matrices and principal coordinates plots generated to visualise relationships between microbiomes based on categories (age, sex, infection status). OTU significance and co-occurrence analysis was performed in QIIME to identify if any OTUs were significantly associated with a category. A paired T-test was used (within R) to compare pre and post-treatment samples for changes in abundance of OTUs; this analysis was carried out for all taxonomic levels. To counteract multiple testing a Bonferroni correction was applied, and a less conservative false discovery rate (FDR) of 0.5.

### Phylogenetic analysis

Representative sequences from OTUs that were significantly different between categories were investigated using more intensive phylogenetic methods. Sequences were imported into the ARB phylogenetic software [30] and aligned against the SILVA reference database LTPs111 [31,32] using the SINA aligner with ARB, and manually checked using the ARB alignment editor. Sequences were inserted into the LTPs111 guide tree using ARB parsimony method, to confirm the taxonomy of these sequences bootstrap values were calculated with the ARB estimation of bootstrap values by parsimony.

## Results

### Cohort infection characteristics before treatment

The139 children (73 male and 66 female) aged 6 months to 13 years had an infection prevalence of 27.3% and a mean infection intensity of 10.17 eggs/10 ml urine (SEM = 2.64; range 0–221). All children were offered treatment with PZQ and of the 139 children 62 were followed up. Of these 62 children, 47 were treated with PZQ (11 egg positive and, 36 egg negative at baseline) while 15 children who were absent on treatment days or would not accept western treatment on religious grounds, but were willing to continue in the study (4 egg positive and 11 egg negative at baseline) effectively became untreated controls.

### Pre-treatment gut microbiome analysis by host age and sex

**Fig 1:**
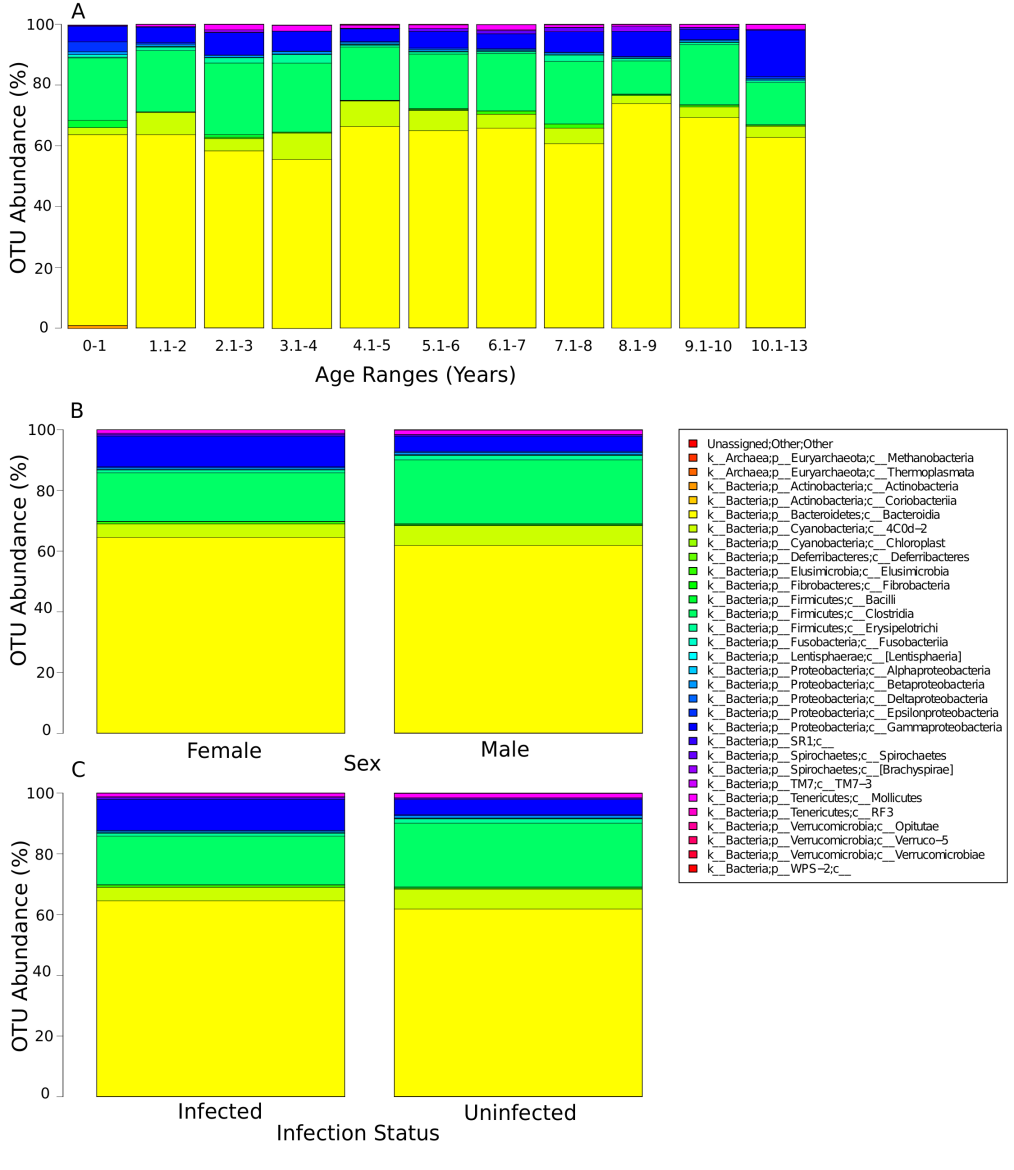
The relative abundance of bacterial classes within the human gut microbiome separated into A) age range, B) sex, C) infection status.

**Fig 2:**
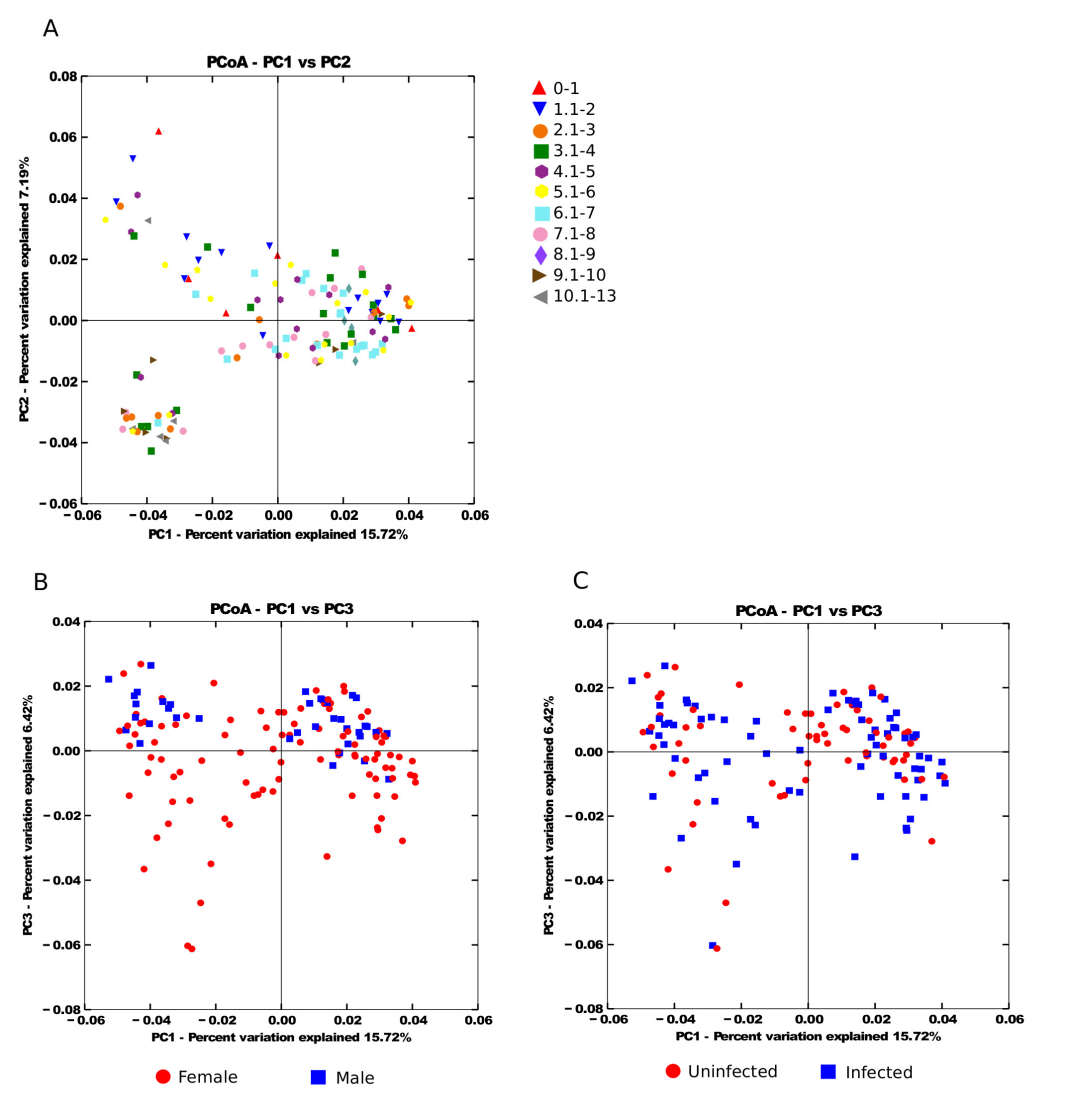
Principal CoOrdinates Analysis of the microbial community similarity by A) age range, B) sex, C) infection status. Distances between samples were calculated using unweighted UniFrac.

Pre-treatment samples were analysed as a separate data set. After filtering and denoising10,722,905 sequences underwent additional analysis (77,143 mean sequences/sample). The data were analysed to determined differences with age group and sex. The relative abundance of taxa among bacterial classes showed limited variation by age group or sex (Fig 1A and 1B). Across all age groups and both sexes, the most abundant phyla observed was Bacteroidetes, followed by Firmicutes, with the least abundant being the Proteobacteria (Table 1). Plots of the first and third principal coordinates showed minimal variation between sex and the first two principal coordinates showed minimal variation between age groups suggesting the presence of communities with similar overall compositions (Fig 2A and 2B). Nonetheless, the abundance of 21 OTUs did differ significantly between age groups (S2 Table). OTU703 (*Prevotella copri*) was present in all age ranges except up to one year. A number of assigned OTUs had a higher abundance before one year compared to all other age ranges, including *Veillonella*, *Streptococcu*s, *Bacteroides* and *Helicobacter*.

**Table 1:** Mean relative abundance of three dominant phyla (Bacteroidetes, Firmicutes and Proteobacteria) within each category (age range, infection based on egg count, serological analysis and sex).

		Mean relative abundance (%)		
Category	Sub-category	Bacteroidetes	Firmicutes	Proteobacteria
Age range	0–1	62.8	23.2	9.4
	1.1–2	63.6	21.4	6.5
	2.1–3	58.3	26.1	8.3
	3.1–4	55.6	25.5	7.7
	4.1–5	66.3	18.0	5.1
	5.1–6	64.9	18.8	6.7
	6.1–7	65.7	19.6	5.7
	7.1–8	60.6	22.7	7.7
	8.1–9	73.8	11.8	9.0
	9.1–10	69.2	20.7	4.3
	10.1–13	62.6	14.5	16.6
Infection status by egg count	Infected	64.4	17.0	11.1
	Uninfected	61.7	22.7	6.2
Infection status by serology	Infected	66.8	18.2	6.5
	Uninfected	62.6	22.9	7.9
Sex	Male	62.5	20.6	9.4
	Female	62.6	21.2	6.3

### Pre-treatment gut microbiome analysis by host infection status

We identified 1120 OTUs in the samples collected. There was significant difference in the diversity of the OTU in infected children vs uninfected children, but this was not robust to Bonferroni correction (Table 2). There were differences in the abundance of 27 OTUs between infected and uninfected children, however, only differences in 5 of these, belonging to the genus *Prevotella*, were significant (FDR<0.05). These are detailed in Table 3. The relative abundance of taxa among bacterial classes by infection status is shown in Fig 1C. Fig 2C shows the plot of the first and third principal coordinates indicating variation between infected vs uninfected children, suggesting the presence of communities with different compositions.

**Table 2:** Two-sided student’s t-test output testing the null hypothesis that there is no difference in OTU diversity for the different age, infection status, and sex categories.

Group 1	Group 2	t statistic	Parametric p-value	Parametric p-value (Bonferroni-corrected)
All within age range	All between age range	-4.184904082	<0.001	0.07
All within infection by egg count	infected vs. uninfected	2.477250355	0.01	0.1
All within Sex	male vs. female	0.423373207	0.67	1

**Table 3:** OTUs whose abundance is significantly higher in schistosome infected children compared to uninfected children. OTU significance was calculated using the ANOVA test after false discovery rate correction (p values <0.05).

OTU	FDR_corrected	Infected _mean	Uninfected_mean	Consensus Lineage
31	0.001274659	451.3947368	102.4081633	*Prevotella*
1098	0.004130435	2.368421053	0.387755102	*Prevotella*
712	0.004130435	35.10526316	9.326530612	*Prevotella*
557	0.049471422	79.86842105	35.71428571	*Prevotella*
500	0.049471422	71.73684211	26.31632653	*Prevotella*

### Effect of curative antihelminthic treatment on the gut microbiome

The efficacy of PZQ treatment of *S*. *haematobium* infection is typically checked 12 weeks after PZQ administration as this period allows the efficacy of treatment to be checked before any re-infections have become patent and started laying eggs [7]. We analysed the baseline (pre-treatment) and post-treatment sequence data from 62 individuals who met the inclusion criteria for the cohort study. After filtering and denoising, we analysed 13,408,420 sequences from these participants to investigate the effect of PZQ on the gut microbiome. We tested the null hypotheses that the abundance and the diversity of the gut microbiome did not differ before and 12 weeks after antihelminthic treatment. The data were analysed using a paired t test, taking into account the multiple comparisons. Paired samples were separated into four categories; infected/treated (n = 11, with 1,807,733 sequences), infected/untreated (n = 4, with 1,213,511 sequences), uninfected/treated (n = 36, with 7,175,780 sequences) and uninfected/untreated (n = 11, 2,495,854 sequences). For all 4 categories no OTUs were found to differ in abundance between the groups at any taxonomic level meaning that antihelminthic treatment PZQ did not alter the gut microbiome structure.

## Discussion

Helminth infections continue to be of major public health significance in child health and development in sub-Saharan Africa. Urogenital schistosomiasis is responsible for the largest schistosomiasis disease burden in this region. In a recent review [4], we suggested that manipulation of the human gut microbiome may offer the potential to improve the outcome of helminth infection. Before that becomes reality there is need for extensive studies establishing, association, causality and mechanisms of interaction between the gut microbiome and helminth infection/pathology. In this study, we investigated the association between *S*. *haematobium* infection status and the gut microbiome structure.

Our results showed that there were significant differences in the gut structure of bacterial species in schistosome infected vs. uninfected children (whether infection is determined via egg counts or serology). We identified 27 OTUs that were more abundant in infected children compared to uninfected children and the principal coordinates plots showed different species compositions between the infection groups. The reasons for these differences have yet to be determined, but we can exclude differences in water contact sites between infected and uninfected children since the water sources for both groups overlapped (as determined by questionnaires administered to the carers of young children and to the older school children). The possible mechanisms of interaction between *S*. *haematobium* and the gut microbiome include direct effects in the gut as *S*. *haematobium* adults can be present in both the pelvic venous plexus and mesenteric veins where females lay eggs found in the lower urinary tract, pelvic organs and gut [33]. However, this is unlikely to be the explanation in this study since none of the children were excreting *S*. *haematobium* eggs in their stool (or for that matter, *S*. *mansoni* or any STH eggs) indicating that the children did not have a helminth infection depositing eggs in the GIT. This suggest that the interaction if any, between the gut microbiome and *S*. *haematobium* infection may be remote through indirect systemic effects as has been suggested for intestinal helminths [34].

A recent study in Asians exposed to intestinal nematodes also reported a greater OTU abundance and species richness in people infected with the nematodes *Trichuris* and *Ascaris* [19]. Similar to our findings, the more abundant species found in the Asian population belonged to the Paraprevotellacae, and this has been attributed largely to the carbohydrate rich diet of African children [31] since the *Prevotella* break down complex plant polysaccharides such as xylan and cellulose which are prevalent in the African diet.

As has been demonstrated by other studies relating the gut microbiome structure to human health, establishing causation is not easy [35]. Thus, from our study, it is not possible to determine whether the microbiome structure predisposed the children to schistosome infection e.g. by affecting innate immune responses, or if the schistosome infection resulted in alterations of the gut microbiome, or indeed if both the risk of infection and gut microbiome structure respond to a third component, such as host dietary shifts or innate susceptibility. Experimental studies in macaques suffering from idiopathic chronic diarrhoea have shown that helminth infection can restore diversity to the gut microbiome and have begun to elucidate the mechanisms mediating this effect [36]. These include alteration of the structure of the mucosal by activating mucus production and epithelial cell turnover which allow attachment of a diverse population of gut bacteria [36]. The clinical significance of these findings still has yet to be determined e.g. are any of the bacteria occurring in infected but not uninfected children associated with any intestinal pathology?

Several reviews have highlighted the importance of the gut microbiome in child health, metabolism, and immune system development [36,37]. In our study although children are infected early, with the youngest person diagnosed with schistosome infection being less than one year old, as reviewed by several others, the establishment of the gut microbiome is already well underway in almost all subjects [36,37], so it is likely that schistosome infection was superimposed on an existent microbiome structure. It is clear from our results that the microbiome structure is most variable between the ages of six months to three years. While a number of assigned OTUs had similar abundance among all age ranges, including *Lachnospiraceae*, *Faecalibacterium*, *Neisseria*, *Blautia*, *Coprococcus*, *Haemophilus* and *Clostridium XIVb*, there were some age-related differences in the abundance of some OTUs within the six months to three year old age group. Changes in the gut microbiome structure in the first three years of life are expected, as this is the age group when the gut microbiome structure is being established [38]. In this population, similar to most rural populations in the area, the majority of the children are breastfed (as opposed to formula-fed) for at least the first six months and this will impact on their gut microbiome structure [39]. In our study population, some gut bacteria groups associated with breastfeeding [40] e.g. *Veillonella* and *Streptococcus*, were present only in the children aged 1 year and below. The age-related differences in the abundance and species diversity in this population were independent of infection status or host sex. Studies with larger sample sizes will determine if there is an interaction between age and infection status which may elucidate the causal association between gut microbiome structure and schistosome infection.

There were no significant sex-related differences in the children’s gut microbiome. This observation is not surprising given that the children enrolled in the study are young and the majority of them have not reached puberty, where the influences of the sex hormones on physiology or innate immune responses can be marked. Sex-related differences in schistosome infection intensity have often been explained by sex-related differences in patterns of exposure to infective water [41]. However, we did not observe sex-related differences in the gut microbiome structure in infected vs uninfected children. A study with a larger sample size of children including heavily infected children may shed more light on the possible interactive effects of host sex and infection status on the gut microbiome.

The gut microbiome can be affected by antibiotic drugs. The effect of the anti-schistosome drug PZQ on the gut microbiome has not yet been studied. In this study we investigated the effect of PZQ treatment on both schistosome infected and uninfected children as is the practise for the schistosome mass drug administration programmes [25]. We found that the gut microbiome remains stable after a single antihelminthic treatment in both infected and uninfected children. In schistosome infected children, treatment-related diarrhoea (usually associated with infection intensity) occurs within 24 hours of treatment [22]. In this study, the children were surveyed 12 weeks after treatment, thus it seems that any PZQ-diarrhoea induced changes in the gut microbiome are no longer apparent by the 12 week survey point. Since the treatment kills adult worms in infected children, our study shows that removal of the parasites from infected children by the drug treatment did not alter the gut microbiome. This is similar to results from the Ecuadorian children infected with the intestinal helminth *Trichuris trichuria*, who showed no change in their gut microbiome following antihelminthic treatment [42]. Similarly treatment of uninfected children did not alter their gut microbiome structure suggesting that the drug PZQ did not alter the gut microbiome structure. As we surveyed children 12 weeks after treatment, we cannot exclude the possibility that treatment-related diarrhoea, which occurs within 24 hours of treatment, might be accompanied by a transient dysbiosis—a phenomenon unlikely to be of clinical significance. The treatment regimen we used for this study i.e. single dose of PZQ at 40mg/kg body weight with an efficacy check follow-up between 6–12 weeks post-treatment is the standard protocol recommended by the World Health Organisation [25]. Therefore, we can exclude long-term (within 12 weeks) effects of PZQ treatment on the gut microbiome structure using the current treatment regimen. The effects of repeated treatment and over a period longer than 12 weeks have yet to be investigated. This result is timely as preparations are now underway for clinical trials of a paediatric formulation of PZQ. Future studies will incorporate a shorter observation time point to determine the more immediate effects on the gut microbiome.

In conclusion, this is the first study investigating the gut microbe structure in children exposed to schistosome infection. Age related differences occur early but the gut microbiome stabilises by the age of three. Furthermore, we identified differences in the microbiome structure of schistosome infected vs. uninfected children, showing increased gut microbiome abundance and species diversity in infected children. Finally we demonstrated that the currently recommended antihelminthic treatment regimen does not alter the host gut microbiome structure when assessed 12 weeks post-treatment. The study highlights several current knowledge gaps which should become narrower with results from current research efforts.

## Supporting Information

S1 Table Primer used for high-throughput sequencing of V3-V4 16S rRNA gene fragments. (DOCX)

S2 Table Twenty-one OTUs associated with all age ranges. (DOCX)
