# Correction: Differences in the Faecal Microbiome in *Schistosoma haematobium* Infected Children vs. Uninfected Children

**DOI:** 10.1371/journal.pntd.0003969

**Published:** 2015-07-30

**Authors:** The PLOS Neglected Tropical Diseases Staff

The labels on parts B and C of Fig 2 are incorrect. The authors have provided a corrected version with a corrected figure legend here.

**Fig 2:**
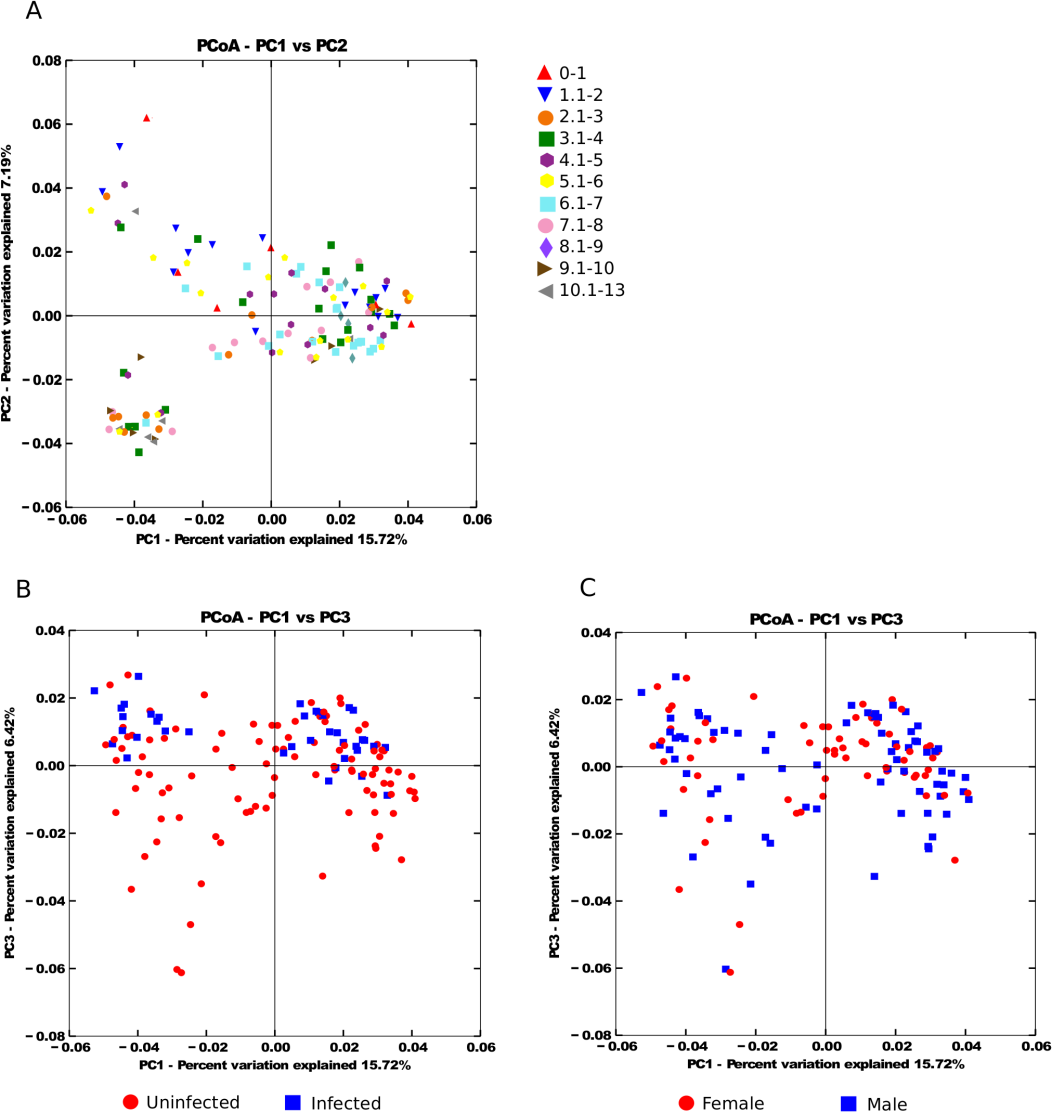
Principal CoOrdinates Analysis of the microbial community similarity by A) age range, B) infection status, C) sex. Distances between samples were calculated using unweighted UniFrac.
