# Supplementary material for: Differences in the Faecal Microbiome in Schistosoma haematobium Infected Children vs. Uninfected Children
Source: PLoS Negl Trop Dis. 2015 Jun 26;9(6):e0003861. doi: 10.1371/journal.pntd.0003861 (PMC4482744; doi:10.1371/journal.pntd.0003861)
Supplement: S1 Table — (DOCX) [file pntd.0003861.s001.docx]

**Table S1: Primer sequences V3-V4 16S rRNA gene**

| primer name | sequence |
| --- | --- |
| 16S_338_P7F1.2 | CAAGCAGAAGACGGCATACGAGATCGTGATGTGACTGGAGTTCAGACGTGTGCTCTTCCGATCT**ACTCCTACGGGDGGCWGCAG** |
| 16S_338_P7F2.2 | CAAGCAGAAGACGGCATACGAGATACATCGGTGACTGGAGTTCAGACGTGTGCTCTTCCGATCT**ACTCCTACGGGDGGCWGCAG** |
| 16S_338_P7F3.2 | CAAGCAGAAGACGGCATACGAGATGCCTAAGTGACTGGAGTTCAGACGTGTGCTCTTCCGATCT**ACTCCTACGGGDGGCWGCAG** |
| 16S_338_P7F4.2 | CAAGCAGAAGACGGCATACGAGATTCCTGAGTGACTGGAGTTCAGACGTGTGCTCTTCCGATCT**ACTCCTACGGGDGGCWGCAG** |
| 16S_338_P7F5.2 | CAAGCAGAAGACGGCATACGAGATCACTGTGTGACTGGAGTTCAGACGTGTGCTCTTCCGATCT**ACTCCTACGGGDGGCWGCAG** |
| 16S_338_P7F6.2 | CAAGCAGAAGACGGCATACGAGATATTGGCGTGACTGGAGTTCAGACGTGTGCTCTTCCGATCT**ACTCCTACGGGDGGCWGCAG** |
| 16S_338_P7F7.2 | CAAGCAGAAGACGGCATACGAGATGATCTGGTGACTGGAGTTCAGACGTGTGCTCTTCCGATCT**ACTCCTACGGGDGGCWGCAG** |
| 16S_338_P7F9.2 | CAAGCAGAAGACGGCATACGAGATCTGATCGTGACTGGAGTTCAGACGTGTGCTCTTCCGATCT**ACTCCTACGGGDGGCWGCAG** |
| 16S_338_P7F10.2 | CAAGCAGAAGACGGCATACGAGATAAGCTAGTGACTGGAGTTCAGACGTGTGCTCTTCCGATCT**ACTCCTACGGGDGGCWGCAG** |
| 16S_338_P7F11.2 | CAAGCAGAAGACGGCATACGAGATGTAGCCGTGACTGGAGTTCAGACGTGTGCTCTTCCGATCT**ACTCCTACGGGDGGCWGCAG** |
| 16S_338_P7F12.2 | CAAGCAGAAGACGGCATACGAGATTACAAGGTGACTGGAGTTCAGACGTGTGCTCTTCCGATCT**ACTCCTACGGGDGGCWGCAG** |
| 16S_338_P7F13.2 | CAAGCAGAAGACGGCATACGAGATCGAAACGTGACTGGAGTTCAGACGTGTGCTCTTCCGATCT**ACTCCTACGGGDGGCWGCAG** |
| 16S_806_P5R2 | AATGATACGGCGACCACCGAGATCTACACTCTTTCCCTACACGACGCTCTTCCGATCTNNNNAGTCAA**GGACTACIRGGGTATCTAAKCC** |
| 16S_806_P5R3 | AATGATACGGCGACCACCGAGATCTACACTCTTTCCCTACACGACGCTCTTCCGATCTNNNNAGTTCA**GGACTACIRGGGTATCTAAKCC** |
| 16S_806_P5R4 | AATGATACGGCGACCACCGAGATCTACACTCTTTCCCTACACGACGCTCTTCCGATCTNNNNATGTCA**GGACTACIRGGGTATCTAAKCC** |
| 16S_806_P5R5 | AATGATACGGCGACCACCGAGATCTACACTCTTTCCCTACACGACGCTCTTCCGATCTNNNNCCGTCC**GGACTACIRGGGTATCTAAKCC** |
| 16S_806_P5R6 | AATGATACGGCGACCACCGAGATCTACACTCTTTCCCTACACGACGCTCTTCCGATCTNNNNGTAGAG**GGACTACIRGGGTATCTAAKCC** |
| 16S_806_P5R7 | AATGATACGGCGACCACCGAGATCTACACTCTTTCCCTACACGACGCTCTTCCGATCTNNNNGTCCGC**GGACTACIRGGGTATCTAAKCC** |
| 16S_806_P5R8 | AATGATACGGCGACCACCGAGATCTACACTCTTTCCCTACACGACGCTCTTCCGATCTNNNNGTGAAA**GGACTACIRGGGTATCTAAKCC** |
| 16S_806_P5R9 | AATGATACGGCGACCACCGAGATCTACACTCTTTCCCTACACGACGCTCTTCCGATCTNNNNGTGGCC**GGACTACIRGGGTATCTAAKCC** |
