# Supplementary material for: Differences in the Faecal Microbiome in Schistosoma haematobium Infected Children vs. Uninfected Children
Source: PLoS Negl Trop Dis. 2015 Jun 26;9(6):e0003861. doi: 10.1371/journal.pntd.0003861 (PMC4482744; doi:10.1371/journal.pntd.0003861)
Supplement: S2 Table — (DOCX) [file pntd.0003861.s002.docx]

**Supplementary Table 2:** Significant OTUs associated with age range. OTU significance was calculated using the ANOVA test after false discovery rate correction (p values <0.05).

| OTU | Test-Statistic | P | FDR_P | Bonferroni_P | 0-1 | 1.1-2 | 2.1-3 | 3.1-4 | 4.1-5 | 5.1-6 | 6.1-7 | 7.1-8 | 8.1-9 | 9.1-10 | 10.1-13 | Taxonomy |
| --- | --- | --- | --- | --- | --- | --- | --- | --- | --- | --- | --- | --- | --- | --- | --- | --- |
| 677 | 4.044117647 | 8.01E-05 | 0.006836643 | 0.088876354 | 0 | 0 | 0 | 0 | 0 | 0 | 0 | 0 | 5.25 | 0 | 0 | k__Bacteria;p__Bacteroidetes;c__Bacteroidia;o__Bacteroidales;f__S24-7;g__;s__ |
| 472 | 3.950579381 | 0.000106937 | 0.008470959 | 0.118593423 | 0 | 0.5 | 0.083333333 | 0.263157895 | 2.785714286 | 0.333333333 | 0.285714286 | 0.153846154 | 85.25 | 0 | 0 | k__Bacteria;p__Cyanobacteria;c__4C0d-2;o__YS2;f__;g__;s__ |
| 703 | 3.616764936 | 0.000299611 | 0.020766773 | 0.332268372 | 0 | 1.3125 | 161.1666667 | 66.05263158 | 29.42857143 | 60.66666667 | 22.47619048 | 92.23076923 | 5.75 | 246.5714286 | 221.3333333 | k__Bacteria;p__Bacteroidetes;c__Bacteroidia;o__Bacteroidales;f__Prevotellaceae;g__Prevotella;s__copri |
| 451 | 3.574555072 | 0.000341291 | 0.022264223 | 0.378491796 | 0.166666667 | 3.875 | 0.833333333 | 1.368421053 | 0.142857143 | 0.388888889 | 0 | 0.076923077 | 0 | 0.142857143 | 0 | k__Bacteria;p__Firmicutes;c__Clostridia;o__Clostridiales;f__Clostridiaceae;g__Clostridium;s__ |
| 341 | 3.837153936 | 0.00015175 | 0.011219365 | 0.168290482 | 0.5 | 0.875 | 3.25 | 2.631578947 | 3.571428571 | 5.166666667 | 8.238095238 | 9.153846154 | 4 | 10.42857143 | 6.166666667 | k__Bacteria;p__Firmicutes;c__Clostridia;o__Clostridiales;f__Ruminococcaceae;g__;s__ |
| 1008 | 6.914136622 | 1.51E-08 | 5.57E-06 | 1.67E-05 | 1.5 | 0.125 | 0 | 0 | 0 | 0 | 0 | 0 | 0 | 0 | 0 | k__Bacteria;p__Firmicutes;c__Clostridia;o__Clostridiales;f__Clostridiaceae;g__Clostridium;s__neonatale |
| 832 | 3.555204686 | 0.000362287 | 0.022320931 | 0.40177676 | 1.5 | 0 | 0 | 0.157894737 | 0.071428571 | 0 | 0 | 0.153846154 | 0 | 0 | 0 | k__Bacteria;p__Proteobacteria;c__Betaproteobacteria;o__Burkholderiales;f__Oxalobacteraceae;g__;s__ |
| 797 | 5.067790732 | 3.50E-06 | 0.000553816 | 0.003876711 | 2.666666667 | 0.125 | 0.083333333 | 0 | 0 | 0.055555556 | 0 | 0 | 0 | 0 | 0 | k__Bacteria;p__Firmicutes;c__Clostridia;o__Clostridiales;f__Veillonellaceae;g__Veillonella;s__ |
| 628 | 3.278380386 | 0.000850098 | 0.044893271 | 0.942758695 | 3.333333333 | 0.5625 | 0.666666667 | 0.105263158 | 0 | 0.055555556 | 0.095238095 | 0.153846154 | 0.25 | 0 | 0 | k__Bacteria;p__Firmicutes;c__Clostridia;o__Clostridiales;f__Veillonellaceae;g__Megasphaera;s__ |
| 382 | 5.226504005 | 2.16E-06 | 0.000399896 | 0.002399377 | 15.83333333 | 0.125 | 0 | 0 | 0 | 0 | 0 | 0 | 0 | 0 | 0 | k__Bacteria;p__Bacteroidetes;c__Bacteroidia;o__Bacteroidales;f__Bacteroidaceae;g__Bacteroides;s__ |
| 31 | 3.451146244 | 0.000499372 | 0.029147577 | 0.553803962 | 26.83333333 | 10.4375 | 93.16666667 | 93.63157895 | 137.1428571 | 248.3888889 | 310.7619048 | 146.1538462 | 143.75 | 548.2857143 | 789 | k__Bacteria;p__Bacteroidetes;c__Bacteroidia;o__Bacteroidales;f__Prevotellaceae;g__Prevotella;s__ |
| 534 | 6.952620113 | 1.35E-08 | 5.57E-06 | 1.50E-05 | 36 | 3 | 0.583333333 | 1.157894737 | 0 | 2.666666667 | 0 | 2 | 0 | 0 | 0 | k__Bacteria;p__Actinobacteria;c__Actinobacteria;o__Bifidobacteriales;f__Bifidobacteriaceae;g__Bifidobacterium;s__ |
| 523 | 5.019539415 | 4.05E-06 | 0.000560893 | 0.004487147 | 52.33333333 | 0 | 0.25 | 0 | 0 | 0 | 0 | 0 | 0 | 0 | 0 | k__Bacteria;p__Firmicutes;c__Clostridia;o__Clostridiales;f__Veillonellaceae;g__Veillonella;s__ |
| 441 | 4.283500475 | 3.83E-05 | 0.00386619 | 0.042528093 | 77 | 3.8125 | 1.083333333 | 2.631578947 | 2.428571429 | 1.888888889 | 1.952380952 | 2.230769231 | 1.5 | 1.285714286 | 0.666666667 | k__Bacteria;p__Firmicutes;c__Bacilli;o__Lactobacillales;f__Streptococcaceae;g__Streptococcus;s__ |
| 209 | 3.341140226 | 0.000700814 | 0.038860142 | 0.777202836 | 134.3333333 | 66.5 | 0.5 | 0.526315789 | 0 | 1.666666667 | 0 | 0.307692308 | 0 | 0 | 0 | k__Bacteria;p__Fusobacteria;c__Fusobacteriia;o__Fusobacteriales;f__Fusobacteriaceae;g__Fusobacterium;s__ |
| 237 | 4.085296738 | 7.06E-05 | 0.006523539 | 0.078282462 | 138.5 | 0.125 | 0.166666667 | 0 | 0 | 0 | 0 | 0.076923077 | 0 | 0 | 0 | k__Bacteria;p__Proteobacteria;c__Epsilonproteobacteria;o__Campylobacterales;f__Helicobacteraceae;g__Helicobacter |
| 45 | 5.432071813 | 1.17E-06 | 0.000258532 | 0.001292658 | 278.5 | 18.8125 | 15 | 9.684210526 | 6.142857143 | 6.111111111 | 5.80952381 | 4 | 5.75 | 10.71428571 | 5.166666667 | k__Bacteria;p__Firmicutes;c__Bacilli;o__Lactobacillales;f__Streptococcaceae;g__Streptococcus;s__ |
| 187 | 12.05246395 | 2.19E-14 | 2.43E-11 | 2.43E-11 | 322.5 | 46.0625 | 38.58333333 | 12.26315789 | 18.07142857 | 7.111111111 | 10 | 14.07692308 | 11 | 9.714285714 | 3.166666667 | k__Bacteria;p__Firmicutes;c__Clostridia;o__Clostridiales;f__Veillonellaceae;g__Veillonella;s__dispar |
| 85 | 4.458060403 | 2.24E-05 | 0.002488064 | 0.024880639 | 374.1666667 | 81.9375 | 47.5 | 41.63157895 | 15.57142857 | 34.16666667 | 24.42857143 | 20 | 30.75 | 117.2857143 | 16.83333333 | k__Bacteria;p__Proteobacteria;c__Gammaproteobacteria;o__Pasteurellales;f__Pasteurellaceae;g__Haemophilus;s__parainfluenzae |
| 16 | 6.746255136 | 2.44E-08 | 6.76E-06 | 2.71E-05 | 653.6666667 | 123.3125 | 88.91666667 | 37.68421053 | 39.57142857 | 32.33333333 | 61.14285714 | 41.07692308 | 44.75 | 56.14285714 | 13.5 | k__Bacteria;p__Firmicutes;c__Clostridia;o__Clostridiales;f__Veillonellaceae;g__Veillonella;s__dispar |
| 57 | 4.869696798 | 6.38E-06 | 0.000785961 | 0.007073647 | 714.3333333 | 237.5625 | 59.41666667 | 59.21052632 | 34.64285714 | 67.27777778 | 9.285714286 | 27.69230769 | 65.25 | 7.142857143 | 9.666666667 | k__Bacteria;p__Proteobacteria;c__Gammaproteobacteria;o__Enterobacteriales;f__Enterobacteriaceae;g__;s__ |
